# Supplementary material for: The Management of Pediatric Acute Appendicitis—Survey among Pediatric Surgeons in Romania
Source: Medicina (Kaunas). 2022 Nov 27;58(12):1737. doi: 10.3390/medicina58121737 (PMC9781247; doi:10.3390/medicina58121737)
Supplement: Supplementary file 1 [file medicina-58-01737-s001.zip › medicina-1977654-supplementary.pdf]

medicina

Supplementary material S1. the Management of Pediatric Acute Appendicitis—Survey among Pediatric Surgeons in Romania

Part 1—General questions

What is your actual position?

- □ Consultant
- □ Specialist
- □ Trainee

What type of hospital do you work in?

- □ Pediatric hospital
- □ Adult&pediatric hospital

How many cases of acute appendicitis do you treat every year?

- □ <12
- □ 12–24
- □ 25–50
- □ 51–75
- □ 76–100
- □ >100

Part 2—Diagnosis

How often do you use the following tools in the diagnosis of acute appendicitis?

	Never (0%)	Rarely (1-33%)	Occasionally (34-66%)	Frequently (67-99%)	Always (100%)
Complete blood count	□	□	□	□	□
C-reactive protein	□	□	□	□	□
Abdominal ultrasound	□	□	□	□	□
CT scan	□	□	□	□	□
MRI scan	□	□	□	□	□

For the diagnosis of acute appendicitis, do you use any of the following scores?

- □ Pediatric Appendicitis Score (PAS)
- □ Alvarado score
- □ None of the above

Part 3—Management

In case of simple appendicitis, do you decide in favor of conservative or operative approach?

- □ Conservative approach

→ What type of antibiotics do you prescribe (class)? _____

what route? _____

for how many days? _____

Do you prescribe it an

□ outpatient or

□ inpatient basis?

- □ Operative approach

  Do you start antibiotics preoperatively?

  □ No

  □ Yes → which antibiotic (class) ____

  In case of a clinically stable patient, do you perform appendectomy during the night?

  □ No

  □ Yes

  Do you prescribe antibiotics postoperatively?

  □ No

  □ I only prescribe one dose intraoperatively

  → which antibiotics (class) ____

  □ Yes → which antibiotic (class) ____

  for how many days ____

In case of complicated appendicitis do you decide in favor of the following:

- □ Antibiotics only (no appendectomy) → which antibiotics (class) ____

  for how many days ____

- □ Appendectomy only in certain cases
- □ Appendectomy

  In case of a clinically stable patient, do you perform appendectomy during the night?

  □ No

  □ Yes

  Do you start antibiotics preoperatively?

  □ No

  □ Yes → which antibiotic (class) ____

  Do you prescribe antibiotics postoperatively?

  □ No

  □ I only prescribe one dose intraoperatively

  → which antibiotics (class) ____

  □ Yes → which antibiotic (class) ____

  for how many days ____

In case of appendicular mass, you decide in favor of

- □ Operative approach
- □ Conservative approach

  → Which antibiotics (class) ___

  In case of favorable outcome (antibiotherapy) do you perform interval appendectomy?

□ Never	
□ Only in certain cases →	When do you perform the appendectomy?<br>□ In 4 weeks<br>□ In 6 weeks
□ At parents' request →	□ In 2 months<br>□ In 3 months
□ Always →	□ In 6 months

In case of a large appendicular abscess, you decide in favor of

- □ Appendectomy
- □ Imaging guided drainage (US/CT)
- □ Other

Part 4—Surgery

In case of simple appendicitis, what is your preferred approach?

- □ Open approach

  The appendicular stump is:

  - □ Always inverted
  - □ Inverted whenever possible
  - □ Let free
  - □ Let free whenever possible

- □ Laparoscopic approach

  How many ports do you use? ___

  How do you resect the appendix?

  - □ Endo-loop
  - □ Stapler
  - □ Suture material—intracorporeal knot
  - □ Suture material—extracorporeal knot

  Do you use an endobag to retrieve the appendix?

  - □ Always
  - □ In certain cases
  - □ Never

In case of complicated appendicitis, what is your preferred approach?

- □ Open approach

  The appendicular stump is:

  - □ Always inverted
  - □ Inverted whenever possible
  - □ Let free
  - □ Let free whenever possible

- □ Laparoscopic approach

  How many ports do you use? ____

  How do you resect the appendix?

- □ Endo-loop
- □ Stapler
- □ Suture material—intracorporeal knot
- □ Suture material—extracorporeal knot

Do you use an endo-bag to retrieve the appendix?

- □ Always
- □ In certain cases
- □ Never

Regardless of the type of appendicitis (simple or complicated)

The appendix is sent of histological analysis

- □ Always
- □ In certain cases
- □ Never

In case of contaminated peritoneal cavity:

Do you send the pus for microbiological analysis?

- □ Always
- □ In certain cases
- □ Never

Do you place a drainage?

- □ Always
- □ In certain cases
- □ Never

Do you irrigate the peritoneal cavity?

- □ Never
- □ Always
- □ Only if it is contaminated

→ If Always/Only if it is contaminated answers:

What do you use?

- □ Normal saline
- □ Normal saline + antibiotics
- □ Other

Part 5—Postoperative management

In case of a patient with simple appendicitis who underwent appendectomy, do you prescribe antibiotics at discharge?

- □ No
- □ Yes → which antibiotic (class) ____
  for how many days ____

Do you follow-up the patients with simple appendicitis who underwent appendectomy?

- ◻ No
- ◻ Yes, for 7 days
- ◻ Yes, for 1 month
- ◻ Yes, for 6 month
- ◻ Yes, for 1 year
- ◻ Yes, for more than 1 year

In case of a patient with complicated appendicitis who underwent appendectomy, do you prescribe antibiotics at discharge?

- ◻ No
- ◻ Yes → which antibiotic (class) ____
  for how many days ____

Do you follow-up the patients with complicated appendicitis who underwent appendectomy?

- ◻ No
- ◻ Yes, for 7 days
- ◻ Yes, for 1 month
- ◻ Yes, for 6 month
- ◻ Yes, for 1 year
- ◻ Yes, for more than 1 year

Varia

Is there any guidelines for antibiotics use in your center?

- ◻ Yes
- ◻ No

Is there any Antibiotic Stewardship program in your center?

- ◻ Yes
- ◻ No
